# *Lannea coromandelica* (Houtt.) Merr. Induces Heme Oxygenase 1 (HO-1) Expression and Reduces Oxidative Stress via the p38/c-Jun N-Terminal Kinase–Nuclear Factor Erythroid 2-Related Factor 2 (p38/JNK–NRF2)-Mediated Antioxidant Pathway

**DOI:** 10.3390/ijms18020266

**Published:** 2017-01-29

**Authors:** Md Badrul Alam, Kyoo-Ri Kwon, Seok-Hyun Lee, Sang-Han Lee

**Affiliations:** 1 Department of Food Science and Biotechnology, Graduate School, Kyungpook National University, Daegu 41566, Korea; mbalam@knu.ac.kr (M.B.A.); rbfl0116@daum.net (K.-R.K.); sam4408@naver.com (S.-H.L.); 2 Food and Bio-Industry Research Institute, Kyungpook National University, Daegu 41566, Korea

**Keywords:** antioxidant, *Lannea coromandelica*, heme oxygenase 1 (HO-1), nuclear factor erythroid 2-related factor 2 (NRF2)

## Abstract

The leaves of *Lannea coromandelica* (Houtt.) Merr. are used in the Garo, Pahan, and Teli tribal communities of Bangladesh as a traditional medicinal plant to treat hepatitis, diabetes, ulcers, heart disease, and dysentery. However, there have been limited phytochemical and biological studies on the bark of *L. coromandelica*. This study aimed to investigate the antioxidant activities of *L. coromandelica* bark extract (LCBE) and the underlying mechanism using RAW 264.7 cells. The LCBE was analysed by high-pressure liquid chromatography (HPLC) to detect its key polyphenolic compounds. Various in vitro antioxidant assays were performed using RAW 264.7 cells to assess the antioxidant effects of the LCBE and to understand the underlying molecular mechanism. HPLC revealed the presence of gallic acid, (−)-epigallocatechin-3-gallate, catechin, chlorogenic acid, and caffeic acid in the LCBE. The extract showed a very potent capacity to scavenge numerous free radicals through hydrogen atom transfer and/or electron donation and also quenched cellular reactive oxygen species (ROS) generation without showing any toxicity. The LCBE was found to combat the oxidative stress by enhancing the expression, at both transcriptional and translational levels, of primary antioxidant enzymes as well as phase II detoxifying enzymes, especially heme oxygenase 1, through the upregulation of the nuclear factor erythroid 2-related factor 2 (NRF2)-mediated pathway in RAW 264.7 cells via the phosphorylation of p38 kinase and c-Jun N-terminal kinase (JNK). The LCBE exhibited strong antioxidant activities and mitigated the cellular ROS production. These results provide scientific evidence of its potential as an ideal applicant for a cost-effective, readily available, and natural phytochemical, as well as a strategy for preventing diseases associated with oxidative stress and attenuating disease progress.

## 1. Introduction

Oxidative stress, induced by the production of reactive oxygen species (ROS), including free radical compounds such as the hydroxyl radical (HO^•^) and superoxide anion radical (O_2_^•−^), as well as non-free radical substances such as singlet oxygen (^1^O_2_), hydrogen peroxide (H_2_O_2_), and organic peroxide (ROOH), is considered a major causative factor of a number of physiological disorders. The role of oxidative stress has been confirmed in the development of neurodegenerative disorders (e.g., Alzheimer’s disease and Parkinson’s disease), autoimmune pathologies, diabetes, cancer, and digestive diseases such as gastrointestinal inflammation and ulcerative disorders [1]. In response to these disorders, eukaryotic cells have developed complex signalling cascades such as the induction of cytoprotective and detoxifying enzymes consisting of phase I (cytochrome P_450_) and phase II (detoxifying and antioxidant proteins) enzymes to detoxify potentially detrimental substances and sustain cellular redox homeostasis [2].

The ubiquitously expressed transcription factor nuclear transcription factor erythroid 2-related factor 2 (NRF2) serves as a master regulator of cellular defence against oxidative stress by modulating the basal and inducible expression of various detoxifying and antioxidant enzymes, such as heme oxygenase 1 (HO-1), NAD(P)H:quinone oxidoreductase (NQO1), glutathione *S*-transferase (GST), and γ-glutamyl cysteine synthetase catalytic subunit (GCLc), which are associated with oxidative stress response, cytoprotection, and drug metabolism [3]. Under physiological conditions, NRF2 is inactive; it is sequestered in the cytoplasm by Kelch-like enoyl-coenzyme A hydratase-associated protein 1 (KEAP1), which targets the NRF2 protein for proteasome degradation and ubiquitination and suppresses its translocation into the nucleus [4]. A conformational change of KEAP1 through interactions with various inducers releases NRF2, which translocates to the nucleus and binds to antioxidant-related elements (AREs) in the promoter regions of cytoprotective and antioxidant genes [5]. In addition, several signalling cascades mediated by mitogen-activated protein kinases (MAPKs), such as c-Jun N-terminal kinase (JNK) and extracellular signal-regulated kinase (ERK), as well as by protein kinase C and phosphatidylinositol 3-kinase (PI3K/AKT), are involved in the phosphorylation of NRF2 and its translocation into the nucleus [6].

Antioxidants, which modulate oxidative processes in the body, are believed to be important for health maintenance. Even though many synthetic antioxidants are available and frequently used, the safety and toxicity of these antioxidants have raised imperative concerns [7]. However, their natural counterparts, which have low toxicity and side-effect profiles, have been under extensive consideration for the development of effective and safe drugs and dietary molecules [8]. Therefore, in recent years, antioxidants of phytochemical origin have seen an unprecedented demand as biopharmaceuticals and nutraceuticals, in addition to their use as food additives.

The genus *Lannea* belongs to the family Anacardiaceae and includes tropical deciduous trees widely distributed in India, Bangladesh, and other tropical countries. Traditionally, various parts of the plants have been used as a medicine for various diseases. The stem bark is used by the Garo, Pahan, and Teli tribal communities in Bangladesh to treat hepatitis, diabetes, ulcers, heart disease, and dysentery [9]. Leaf juice is orally taken to alleviate ulcers and pain [10], while the sap of the fruit is used to treat cold and cough [11]. The bark is used for gout, dyspepsia, dysentery [12], eruption of the skin, ulcers, and toothache [13]. Based on these traditional applications, researchers have conducted scientific studies to validate the use of *Lannea* spp. against various diseases. It has been shown that twigs induce apoptosis of human liver cancer cells [14] and leaves have antinociceptive [15], antioxidant [16], and antidiarrheal [17] activities. Barks were found to have anti-inflammatory [18], hypotensive [19], antihyperglycaemic [9], antimicrobial, antifungal [7], analgesic, and antioxidant effects [20], and the bark showed zoosporicidal activity [21]. However, no studies have been achieved to date to scrutinize which subset of antioxidant enzymes is transcriptionally controlled by *Lannea coromandelica* (Houtt.) Merr. bark extract (LCBE), and the mechanisms behind the LCBE regulation of the expression of these enzymes are yet to be investigated.

Therefore, we hypothesised that components of LCBE are functionally associated with NRF2 to stimulate the expression of certain antioxidant enzymes. The current study focused on the role of LCBE in cell antioxidant processes by using various in vitro antioxidant assays and measuring the messenger RNA/protein expressions of antioxidant enzymes in RAW 264.7 cells. We also studied the underlying mechanisms by determining the activation of NRF2 functions by LCBE in RAW 264.7 cells. The results revealed that LCBE controls antioxidant enzymes by moderating the NRF2 function in RAW 264.7 cells. 

## 2. Results

### 2.1. High-Pressure Liquid Chromatography (HPLC) Analysis of Lannea coromandelica Bark Extract

**Figure 1:**
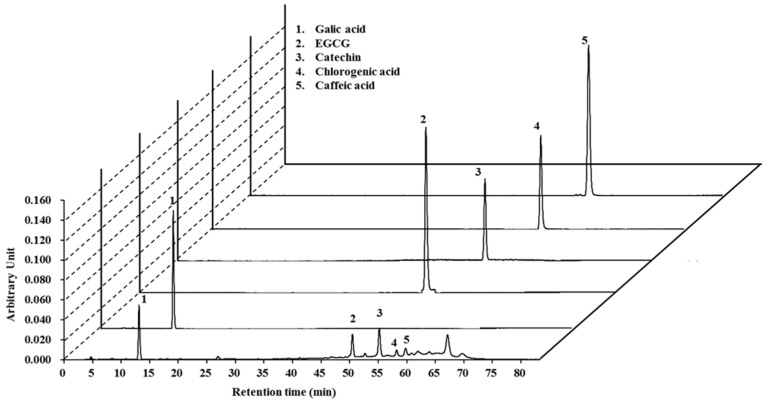
HPLC chromatogram of the *Lannea coromandelica* bark extract with standards, monitored at 280 nm. EGCG: epigallocatechin-3-gallate.

To detect the active components of the LCBE, HPLC analysis was performed using five representative antioxidant standard materials. Interestingly, the LCBE exhibited peaks with the same retention times as the following standard compounds: gallic acid (13.07 min), epigallocatechin-3-gallate (EGCG; 50.38 min), catechin (55.21 min), chlorogenic acid (57.52 min), and caffeic acid (59.69 min) (Figure 1).

### 2.2. Radical-Scavenging Activities of LCB Extract

**Figure 2:**
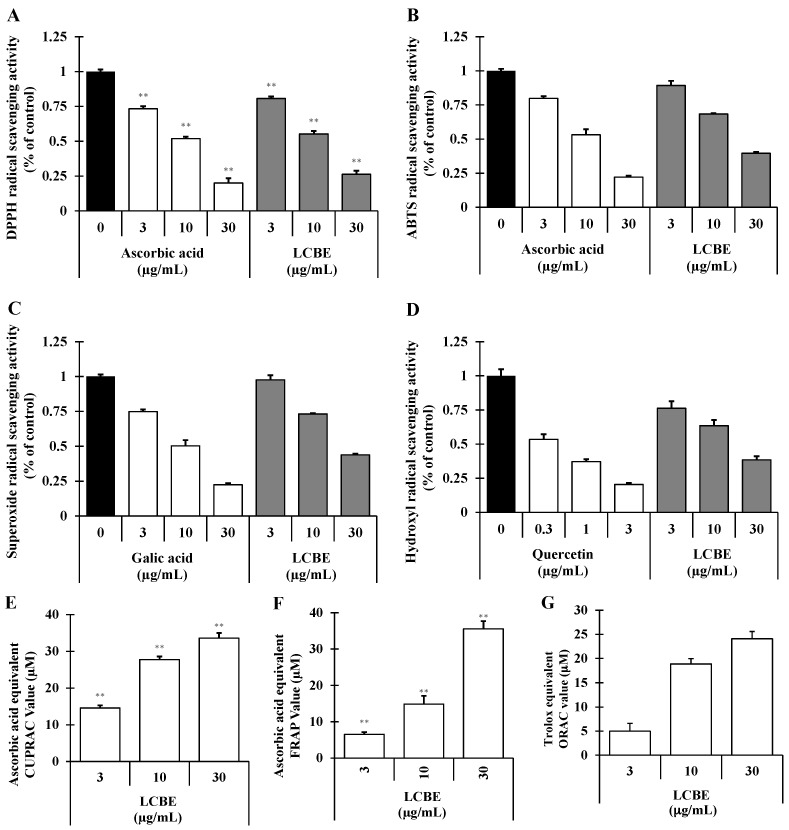
Radical-scavenging effects of *Lannea coromandelica* bark extract (LCBE). The 2,2-diphenyl-1-picrylhydrazyl (DPPH) radical-scavenging assay (**A**), ABTS^•+^ radical-scavenging assay (**B**), superoxide radical-scavenging assay (**C**), hydroxyl (OH) radical-scavenging assay (**D**), cupric reducing antioxidant capacity (CUPRAC) assay (**E**), and ferric reducing antioxidant power (FRAP) assay (**F**) were performed predetermined concentrations of the LCBE, and ascorbic acid, gallic acid, and quercetin were used as positive control. The net area under the curve (net AUC) was used to calculate the oxygen radical absorbance capacity (ORAC) activities (**G**) of the samples. ** *p* < 0.01 versus control using the Student’s *t*-test.

Depending on their mechanisms of action, antioxidants may inhibit cellular damage caused by oxidative stress directly or indirectly. Scavenging of free radicals, ROS, and/or reactive nitrogen species (RNS) by an antioxidant through the donation of hydrogen or electron is considered a direct pathway. In contrast, indirect antioxidant pathways induce the expression of phase II detoxifying and antioxidant genes [22]. To investigate whether LCBE has the direct antioxidant potential with respect to radical-scavenging activities, DPPH^•^-, ABTS^•+^-, superoxide radical-, and hydroxyl radical-scavenging activities were measured. The LCBE scavenged DPPH^•^, a steady organic nitrogen radical, dose-dependently (Figure 2A). Furthermore, in the 2,2′-azino-bis(3-ethylbenzthiazoline-6-sulphonic acid (ABTS) assay, a mixed electron transfer and hydrogen atom transfer assay in which the radical cation is reduced in ABTS^•+^, the LCBE dose-dependently scavenged ABTS^•+^ (Figure 2B). The superoxide radical-scavenging capacity of the LCBE was assessed using the phenazine methosulphate (PMS)–NADH superoxide-generating system, and the results are presented in Figure 2C. Hydroxyl radicals (HO^•^) are extremely sensitive free radicals created in biological systems and capable of damaging almost every molecule found in cells [7]. In this study, the LCBE abolished the production of hydroxyl radicals in the Fenton reaction concentration-dependently (Figure 2D). Additionally, to investigate whether the LCBE has the electron-donating potential, cupric reducing antioxidant capacity (CUPRAC), ferric reducing antioxidant power (FRAP), and oxygen radical absorbance capacity (ORAC) assays were performed, and it was found that the LCBE showed strong and concentration-dependent reducing power capacity (Figure 2E–G). On the basis of these results, we speculated that LCBE has a very strong potentiality to donate electron and/or transfer hydrogen atom to quench free radicals.

### 2.3. LCB Extract Attenuates Cellular Oxidative Stress Induced by 2,2′-Azobis(2-Amidinopropane) Dihydrochloride (AAPH) in RAW 264.7 Cells

**Figure 3:**
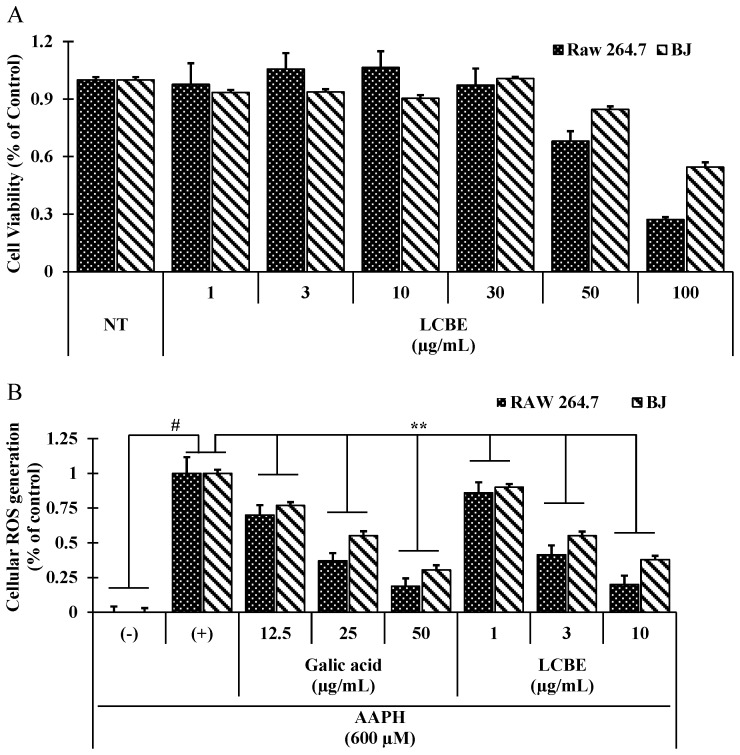
Cell viability and inhibition of reactive oxygen species (ROS) generation in RAW 264.7 cells and BJ cells. RAW 264.7 cells and BJ cells (2 × 10^4^ cells/well) were seeded in 96-well plates, and then the 3-(4,5-dimethylthiazol-2-yl)-2,5-diphenyltetrazolium bromide (MTT) assay (**A**) and DCFH–DA assay (**B**) were conducted with various concentrations of the LCBE. # *p* < 0.001 versus normal control; **p < 0.01 versus 2,2′-azobis(2-amidinopropane) dihydrochloride (AAPH) treatment using the Student’s *t*-test.

Because the LCBE showed strong radical-scavenging activities, it was tested for scavenging activity against cellular ROS induced by 2,2′-azobis(2-amidinopropane) dihydrochloride (AAPH), with gallic acid (a positive control). AAPH is a water-soluble azo small molecule, and upon decomposition, AAPH generates one mole of nitrogen and two moles of carbon radicals. The carbon radicals can either bind to generate secure products or react with molecular oxygen to produce peroxyl radicals, thus continuing a constant rate of free radical production in solution [23]. LCBE treatment mitigated the generation of ROS in AAPH-induced RAW 264.7 cells as well as BJ cells in a dose-dependent manner (Figure 3B), without showing any toxicity (Figure 3A). The relative ROS level in the LCBE (30 µg/mL)-treated Raw 264.7 cells was similar to the ROS-scavenging activity of gallic acid at 50 μg/mL (18.86% ± 2.63% and 18.56% ± 2.33%, respectively), and also LCBE (30 µg/mL) treatment significantly reduced the relative ROS generation (37.95% ± 2.76%) in BJ cells.

### 2.4. Effects of LCB Extract on Antioxidant Enzyme Expression

**Figure 4:**
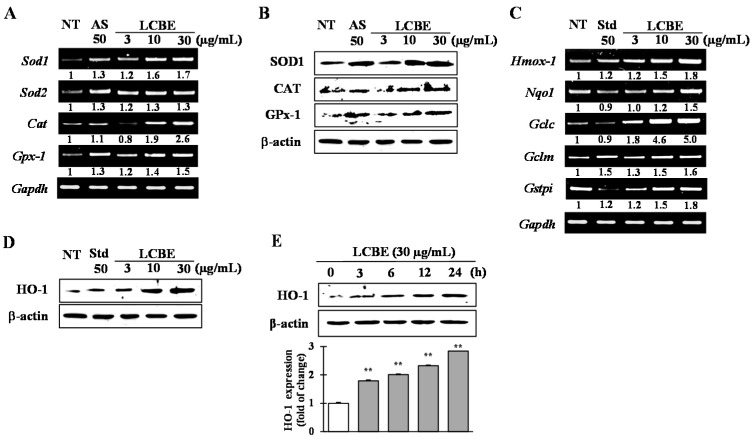
Comparison of expressions of primary- and phase II-antioxidant and detoxifying enzymes. RAW 264.7 cells were pre-treated for 24 h with the specified concentrations of the LCBE. The mRNA expression of the genes for primary antioxidant enzymes (**A**) and phase II antioxidant and detoxifying enzymes (**C**) was measured by reverse transcription-polymerase chain reaction (RT-PCR). The protein levels of primary antioxidant enzymes (**B**) and heme oxygenase 1 (HO-1), regulated by the LCBE in a concentration- (**D**) and time-dependent (**E**) fashion, were determined by western blot. NT: No treatment; AS: Ascorbic acid; Std: epigallocatechin-3-gallate (EGC); SOD: superoxide dismutase; CAT: catalase; GPx: glutathione peroxidase; Nqo1: NAD(P)H:quinone oxidoreductase; Gclc: γ-glutamyl cysteine synthetase catalytic subunit. ** *p* < 0.01 versus control using the Student’s *t*-test.

To investigate the effects of LCBE on the induction of the antioxidant enzymes superoxide dismutase (SOD)1/SOD2, catalase (CAT), and glutathione peroxidase 1 (GPX1), and the phase II detoxifying enzymes HO-1, NQO1, GCLc, GCLm, and GSTpi), RAW 264.7 cells were treated with the LCBE at various concentrations for 24 h. Reverse transcription (RT)-PCR analysis indicated a dose-dependent increase in the mRNA expression of *Sod1*, *Sod2*, *Cat*, and *Gpx1* (Figure 4A), as well as in the expression of the genes encoding phase II detoxifying enzymes, HO-1, NQO1, GCLc, GCLm, and GSTpi (Figure 4C). Increases in the protein levels of SOD1, CAT, GPX1 (Figure 4B), and HO-1 (Figure 4D) were further checked by western blot analysis. In addition, time-dependent western blot analysis revealed significantly enhanced protein expression of HO-1 from 3 to 24 h, peaking at 24 h after LCBE (Figure 4E). These data suggested that the antioxidant activity of the LCBE might be due to an increase in transcriptional and translational expression of primary antioxidant enzymes as well as phase II detoxifying enzymes.

### 2.5. Effects of LCB Extract on Phase II Enzymes through NRF2 Nuclear Translocation

**Figure 5:**
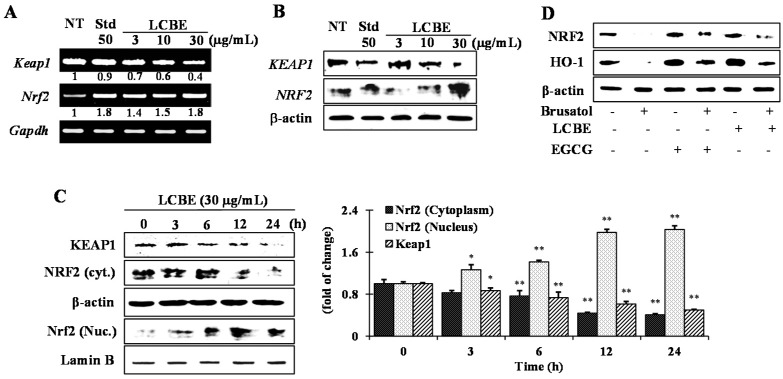
Effect of *Lannea coromandelica* bark extract (LCBE) on translocation of nuclear factor erythroid 2-related factor 2 (NRF2). RAW 264.7 cells were pre-treated for 24 h with the specified concentrations of the LCBE. The mRNA (**A**) and protein (**B**) expression of NRF2 and Kelch-like enoyl-coenzyme A hydratase-associated protein 1 (KEAP1) were determined by reverse transcription-polymerase chain reaction (RT-PCR) and western blot, respectively. The time-dependent changes in the protein levels of nuclear NRF2 were measured by western blot (**C**). Cells were treated with a specific NRF2 inhibitor (brusatol), with and without the LCBE, and the protein levels of NRF2 and HO-1 expression were analysed by western blotting (**D**). NT: No treatment; Std: EGCG as a standard. * *p* < 0.05 and ** *p* < 0.01 versus control using the Student’s *t*-test.

Nearly all phase II detoxifying and antioxidant genes have an ARE sequence in their promoter region, which intercedes the transcriptional up-regulation of these genes [24]. NRF2 is a key transcription factor that controls ARE-driven HO-1 gene expression. Upon induction, NRF2 is considered to dissociate from the KEAP1–NRF2 complex and to translocate into the nucleus where it enhances the expression of phase II genes [25]. Therefore, to elucidate the mechanism of NRF2 activation by LCBE, the mRNA and protein levels of KEAP1 and NRF2 were analysed in the nucleus and cytosol. LCBE treatment increased the mRNA level of *Nrf2*, whereas that of *Keap1* was mitigated dose-dependently (Figure 5A). Additionally, LCBE treatment notably down-regulated the KEAP1 protein expression both dose- (Figure 5B) and time-dependently (Figure 5C). Furthermore, a time course experiment uncovered that the LCBE caused a time-dependent increase in NRF2 translocation into the nucleus, reaching a peak level 12 h of the LCBE treatment (Figure 5C). Based on these data, we concluded that LCBE might interrupt the binding of NRF2 to KEAP1, releasing NRF2 for nuclear translocation. Furthermore, to prove that the LCBE activates phase II enzymes through NRF2, cells were treated with brusatol (a specific NRF2 inhibitor) before LCBE treatment. Brusatol caused meaningful inhibition of NRF2 protein levels, which were not affected by the addition of the LCBE (Figure 5D). Furthermore, induction of the HO-1 protein by the LCBE was also effectively attenuated as a result of NRF2 inhibition (Figure 5D). The results suggested that the LCBE induced HO-1 up-regulation via NRF2-mediated signalling.

### 2.6. LCB Extract Activates NRF2 via Phosphorylation of JNK and p38 MAPKs

**Figure 6:**
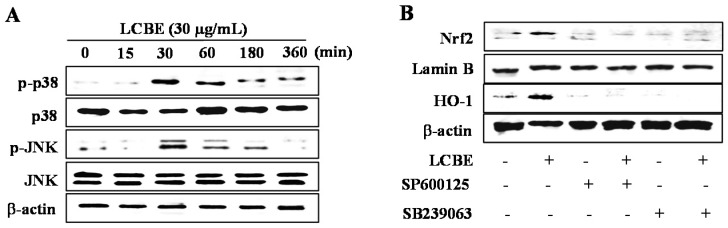
*Lannea coromandelica* bark extract (LCBE) activates the translocation of NRF2 through activation of p38 and c-Jun N-terminal kinase (JNK). RAW 264.7 cells were pre-treated with the LCBE for the indicated periods, and kinase activation was analysed by western blotting (**A**); Cells were treated with a specific inhibitor, SB239063 (a p38 specific inhibitor) and SP600125 (a Jnk specific inhibitor), and the protein levels of NRF2 and HO-1 were analysed by western blotting (**B**).

To investigate the pathways involved in the activation of the NRF2/KEAP1 system, cells were treated with the LCBE for 0.5, 1, 3, 6, and 12 h, and the phosphorylation of ERK1/2, JNK, and p38 was judged by western blotting. As shown in Figure 6A, the LCBE treatment resulted in outstandingly augmented p38 and JNK phosphorylation after 30 min. However, phosphorylated forms of ERK were not found in LCBE-treated cells (data not shown). Therefore, to examine whether the phosphorylation of the p38 and JNK pathways was involved in the induction of NRF2 action and HO-1 expression, specific inhibitors, SB 239063 for p38 and SP600125 for JNK, were applied to cells treated with the LCBE. As expected, the LCBE increased the nuclear NRF2 accumulation and protein expression of HO-1, whereas the inhibition of the p38 and JNK pathways powerfully mitigated these activities of the LCBE (Figure 6B). These results imply that JNK and p38 are associated with the LCBE-induced activation of the NRF2-mediated expression of HO-1 in RAW 264.7 cells.

## 3. Discussion

Oxidative stress can be viewed as an imbalance between the generation of free radicals and their elimination via cellular defence mechanisms. ROS such as the superoxide anion radical (O_2_^•^^−^), hydrogen peroxide (H_2_O_2_), and the hydroxyl radical (HO^•^) are partially produced by the reduction of oxygen during cellular metabolic reactions and served as sever intracellular toxic species. There is considerable evidence that strong, chemically reactive ROS are considered to function entirely as cellular damaging agents, indiscriminately reacting with proteins, lipids, and DNA [26]. In current study, the antioxidant effects of LCBE were assessed using several in vitro chemical assays. It was demonstrated that the essential mechanism by which LCBE mitigates the oxidative stress involves transcriptional and translational regulation of a phase I oxidoreductase and phase II detoxifying enzymes via the KEAP1–NRF2 pathway in RAW 264.7 cells by activating MAPK (p38 and JNK) signalling pathways.

Mounting evidence suggests that phenolic compounds have diverse biological effects, including antioxidant activity. The antioxidant capacity of phenolic compounds is mainly due to their redox capacities, which can play a pivotal role in adsorbing and neutralising free radicals, scavenging singlet and triplet oxygen, and decomposing peroxides [27]. Flavonoids, one of the most diverse and widespread groups of natural compounds, are likely to be the most important natural phenolics and keep a broad spectrum of biological activities, including radical-scavenging properties [28]. Therefore, it was important to determine the total phenolic compounds (247.07 ± 1.02 mg gallic acid equivalent per gram of dry weight) and flavonoids (43.16 ± 1.24 mg catechin equivalent per gram of dry weight) in the plant chosen for this study. The correlation between the content of polyphenols and flavonoids and the antioxidant activity was calculated using the Pearson coefficient (*p*) and linear regression analysis (data not shown). The results exhibited very strong correlations with 2,2-diphenyl-1-picrylhydrazyl (DPPH), ABTS, superoxide, and hydroxyl radical-scavenging activities (*p* = 0.946, 0.962, 0.951, and 0.982, respectively). These data are in accordance with those obtained by other researchers who have displayed that a high total phenol content boosts the antioxidant activities [29] and that there is a linear correlation between the phenolic content and antioxidant activities [30].

The identification of active components in extracts from traditionally used plants is a crucial goal for the development of nutraceuticals. Numerous scientific reports have suggested that *L. coromandelica* is rich in phenol and flavonoid compounds such as ellagic acid, quercetin-3-arabinoside, quercetin [31], (2*R*,3*S*)-(+)-3′,5-dihydroxy-4,7-dimethoxydihydroflavonol, physcion, (2*R*,3*S*)-(+)-4′,5,7-trimethoxydihydroflavonol, (2*R*,3*S*)-(+)-4′,7-di-*O*-methyldihydroquercetin, (2*R*,3*S*)-(+)-4′,7-di-*O*-methyldihydrokaempferol, (2*R*,3*S*)-(+)-4′-*O*-methyldihydroquercetin, leucocyanidin, leucodelphinidin [32], β-sitosterol, and morin E [33]. Based on these data, it was hypothesised that phenolic compounds and flavonoids could be the major components in LCBE since polyphenols and flavonoids normally show a very strong antioxidant potential. To investigate the phytochemicals present in LCBE, HPLC analysis was performed with standard phenolic and flavonoid compounds. It was found that gallic acid, EGCG, catechin, chlorogenic acid, and caffeic acid (Figure 1) were likely present in LCBE, which supported the data from a previous study [16]. Moreover, an in silico investigation was performed to predict the toxic risk assessment as well as biological activities prediction for the major compounds (Table 1 and Table 2). Among the activities predicted by PASS, the presence of mucomembranous protector, fibrinolytic, astringent, antihypercholesterolemic, and hepatoprotectant-like activities support the main traditional uses of *L. coromandelica* for gastrointestinal disorders and diabetes and suggests the identified major compounds as contributors to production of ethnopharmacological effects. Additionally, PASS predicted antioxidant and free radical scavenging properties of EGCG, catechin and chlorogenic acid (probable activity, Pa > 0.8) which were supported by previous studies [34,35]. Furthermore, in silico toxicity of major constituents of LCBE were also evaluated and found very low toxicity probability for LCBE-evaluated major compounds (Table 1).

Because a single method cannot afford an inclusive prediction of the antioxidant potential of various compounds/extracts, a multimethod approach has been proposed to evaluate the antioxidant capacity [36]. Hence, to explore and understand the possible mechanisms, several antioxidant assays, including DPPH^•^-, ABTS^•+^-, superoxide radical-, and hydroxyl radical-scavenging assays as well as FRAP, CUPRAC, and ORAC assays, were performed to estimate the antioxidant activities of LCBE. The results confirmed that LCBE has a broad range of antioxidant properties. The radical-scavenging activities of the LCBE are summarised in Figure 2. The DPPH^•^ and ABTS^•+^ assays are thought to be the most common spectrophotometric methods for the determination of the antioxidant capacity of any extract. The magnitude of free radical quenching in both DPPH and ABTS assays was dose-dependent and steadily increased with the increase of sample concentration (Figure 2A,B). These findings revealed that LCBE has the capacity to act through two different mechanisms to express its free-radical-scavenging activity, a hydrogen transfer reaction (DPPH assay) and a single-electron transfer reaction (ABTS assay) [37]. In contrast, the superoxide anion radical, a weak oxidant, gives rise to dominant and unsafe hydroxyl radicals, as well as to singlet oxygen, both of which contribute to oxidative stress. The LCBE showed a significantly higher tendency to quench the superoxide radical, as indicated by the dose-dependent increase in the percent inhibition (Figure 2C). Furthermore, the hydroxyl radical is a rather reactive ROS, which initiates autooxidation and attacks almost every biological molecule, causing damage to DNA, proteins, and lipids and leading to mutagenesis, carcinogenesis, and aging [38]. Plants with a higher hydroxyl radical-quenching potential are of great importance since their consumption can result in controlling and mitigating the devastating effects of oxidative stress. The current results are of great importance since the LCBE exhibited great potential in scavenging hydroxyl radicals (Figure 2D).

FRAP and CUPRAC are important indicators of the reducing potential of an antioxidant, which is associated with the presence of compounds responsible for the breaking of the free radical chain through donation of the hydrogen atom [39]. Both FRAP and CUPRAC assays provide a reliable method for the evaluation of antioxidant activities of various plant extracts and compounds since their antioxidant capacity is directly correlated with the reducing capacity [7], and our results conform to these findings. The results (Figure 2E,F) demonstrated a noticeable reducing power (ascorbic acid equivalent FRAP and CUPRAC value) of the LCBE, which gradually increased in a concentration-dependent fashion. Furthermore, the ORAC assay is believed to mimic the lipid peroxidantion chain reaction in vivo through the production of AAPH-derived peroxyl radicals, and antioxidant potentiality is measured by assessing the area under the fluorescence decay curve of the inhibition of peroxyl radical-induced oxidation of a fluorescent probe, flurrescein, by antioxidant compounds [40].The net area under the curve (AUC) values of Trolox and LCBE increased in a dose-dependent manner (data not shown), and these results indicated that the LCBE (30 µg/mL) had similar antioxidant capacity to that of 25 mM Trolox (Figure 2G).

The quenching of free radicals, ROS, and RNS through donation of either hydrogen or electrons demonstrates the direct cellular antioxidant capacity of an antioxidant, whereas phase II detoxifying and antioxidant enzymes are believed to provide indirect defensive mechanism against oxidative stress [41]. Free radical and ROS-scavenging activity within cells by enzymes such as SOD, CAT and GPx play a pivotal role in cellular homeostasis during cell proliferation and maintenance. The damage of these key enzymes by continuous oxidative stress can develop in degenerative diseases [42]. Cytosolic superoxide (O_2_^−^) is generated by the one-electron reduction of O_2_ during the mitochondrial electron transport chain reaction. It is well known that O_2_^−^ is rapidly converted into H_2_O_2_ by SOD, and H_2_O_2_ can be detoxified into H_2_O by the scavenging enzymes GPX and CAT. These enzymes act together in the metabolic pathway of free radicals [26,41]. In this study, LCBE treatment significantly increased both mRNA and protein levels of antioxidant enzymes such as SOD1, CAT, and GPX1 (Figure 4A,B) in RAW 264.7 cells and revealed that LCBE has the ability to maintain cellular homeostasis and to protect the cell from oxidative stress.

Following the treatment of RAW 264.7 cells with LCBE for various times and at different doses, significant increases in phase II enzymes were observed at both mRNA and protein levels (Figure 4C–E). GCLc and GCLm are the main enzymes that catalyse glutathione synthesis, which has been shown to decline with the progression of various diseases, such as cancer, obesity, diabetes, neurodegenerative diseases, and age-related macular degeneration [43]. Bilirubin, a strong antioxidant, is generated from heme by the activation of HO-1, encoded by *Hmox1* gene, and HO-1 levels have been found to decrease with increasing oxidative stress [44]. Several nutrients showing benefits against oxidative stress, such as curcumin, caffeic acid ester, eckol, and hydroxytyrosol, have been reported to induce HO-1 expression [45] and our in silico data also support that LCBE have the capacity to enhance the *Hmox1* expression due to the presence of gallic acid, EGCG and catechin. Therefore, it is assumed that the induction of phase II enzymes may be another mechanism accounting for the activity of LCBE against oxidative stress. To further investigate this mechanism, the mRNA and protein levels of NRF2, the key regulator of phase II enzyme activation, were examined. NRF2 is sequestered by KEAP1 in the cytosol and constitutively targeted for polyubiquitination under normal conditions. Cell exposure to electrophilic and oxidative stress, however, releases NRF2 from the KEAP1 complex, which enables NRF2 to translocate to the nucleus and activate a battery of phase II cytoprotective genes by binding to the ARE [46]. In this study, inhibition of NRF2 by a specific inhibitor, brusatol, showed that LCBE mitigated the induction of HO-1 (Figure 5D). This result confirmed previous conclusions regarding the regulation of phase II enzymes by NRF2. Furthermore, both NRF2 and KEAP1 protein levels were regulated by LCBE treatment (Figure 5B). However, after 12 h, LCBE treatment promoted the NRF2 nuclear translocation, accompanied by a decrease in the cytosolic NRF2 content (Figure 5C). Moreover, the treatment with LCBE for various times and at different doses significantly downregulated KEAP1, suggesting that LCBE could activate phase II enzymes through the disruption of the binding of NRF2 to KEAP1. Therefore, it was postulated that the mechanism of interaction between LCBE and the NRF2–KEAP1 complex might mimic the action of other NRF2 inducers, such as *Ginkgo biloba* extract; however, details of the likely interaction between LCBE and KEAP1 require further investigation. *G. biloba* extract was found to stimulate NRF2 and its translocation into the nucleus, with a subsequent increase in the expression of phase II genes, such as *Nqo1*, *Hmox1*, and *Gclc* through the KEAP1–NRF2–ARE signalling pathway in Hepa-1c1c7 and HepG2 cells [24]. An active compound in coffee, 5-*O*-caffeoylquinic acid modulates the NRF2 nuclear translocation and ARE-dependent gene expression, such as *Hmox1*, *Nqo1*, and *Gst* in HT29 cells [47]. Other signalling pathways may also be involved in the regulation of phase II genes or interact with the KEAP1/NRF2/ARE system. The PI3K/AKT and MAPK pathways, including ERK, JNK, and p38 activation, have both been suggested as upstream regulators of NRF2 [48,49]. In the present study, activation of MAPK pathways (JNK and p38) by LCBE was observed over periods ranging from 15 to 360 min. Surprisingly, p38 and JNK inhibitors (SB239063 and SP600125, respectively) effectively inhibited the expression of NRF2 and HO-1 induced by LCBE. Therefore, it was concluded that the NRF2 activation induced by LCBE depends on the activation of the JNK-p38 pathways. Numerous scientific reports have revealed that polyphenolic compounds, such as gallic acid [50], EGCG [34], and chlorogenic acid derivatives [35], have the potential to induce HO-1 expression through NRF2 activation and that the activation of MAPK pathways plays a key role in this mechanism. In this study, HPLC analysis confirmed that gallic acid, EGCG, chlorogenic acid, caffeic acid, and catechin are likely to be present in LCBE and may play the central role in combating the oxidative stress through activation of the JNK/p38–NRF2–HO-1 signalling pathway.

## 4. Experimental Section

### 4.1. Plant Material and Extraction

*L. coromandelica* bark was collected from the adjoining area of the Jahangirnagar University campus, Bangladesh, during August 2015. The plant material was taxonomically identified by the National Herbarium of Bangladesh, whose voucher specimen (DACB: 37549) has been deposited in our laboratory for future reference. Dried and crudely pulverized bark (100 g) was extracted with methanol under reflux for 3 h (three times). The extract was then filtered through filter paper (No. 1 Whatman Schleicher Schuell, Keene, NH, USA). The filtrate was collected and subjected to lyophilisation to give 3.44 g of extract (yield: 3.44%). Finally, the lyophilised sample was dissolved in deionised water at a concentration of 10 mg/mL.

### 4.2. Drugs and Chemicals

2,2-Diphenyl-1-picrylhydrazyl (DPPH), 2,2′-azobis(2-amidinopropane) dihydrochloride (AAPH), phenazine methosulphate (PMS), neocaprione, 2,4,6-tris(2-pyridyl)-*S*-triazine, 2,2′-azino-bis(3-ethylbenzthiazoline-6-sulphonic acid (ABTS), 3-(4,5-dimethylthiazol-2-yl)-2,5-diphenyltetrazolium bromide (MTT), dimethyl sulphoxide (DMSO), 6-hydroxy-2,5,7,8-tetramethylchroman-2-carboxylic acid (Trolox), 2′,7′-dichlorofluorescin diacetate (DCFH-DA), and phosphate-buffered saline (PBS, pH 7.4) were obtained from Sigma-Aldrich (St. Louis, MO, USA). Dulbecco’s modified Eagle’s medium (DMEM), foetal bovine serum (FBS), penicillin-streptomycin mixture, and 0.25% trypsin-ethylenediaminetetraacetic acid (EDTA) were purchased from Gibco BRL Life Technologies (Grand Island, NY, USA). Anti-superoxide dismutase 1 (SOD1), anti-HO-1, anti-catalase (CAT), anti-glutathione peroxidase 1 (GPX1), anti-NRF2, and anti-β-actin were obtained from Santa Cruz Biotechnology (Santa Cruz, CA, USA). Anti-phospho-JNK, anti-p44/42 MAPK (ERK1/2), anti-phospho-p44/42 MAPK (ERK1/2), anti-phospho-p38, and anti-p38 antibodies were also used for this experiment after purchasing from Cell Signaling Technology (Beverly, MA, USA).

### 4.3. High-Pressure Liquid Chromatography with Diode-Array Detection Analysis

Phytochemical characteristics of the LCBE were studied by high-pressure liquid chromatography (HPLC) using standard compounds, including gallic acid, (−)-epigallocatechin gallate (EGCG), catechin, caffeic acid, and chlorogenic acid. HPLC analysis was carried out using an Agilent 1200 chromatographic system (Agilent Technologies, Santa Clara, CA, USA) with a UV-Vis diode-array detector and the ChemStation software (version G2170BA, Agilent Technologies). A 0.45-μm nylon filter (E0034, Análisis Vínicos, Tomelloso, Spain) was used to filter the sample and polyphenolic compounds were analysed according to the method described previously [51]. A Zorbax C18 column (250 × 4.6 mm, 5-μm particle size; Agilent Technologies) was maintained at 30 °C, and elution was performed with a linear gradient of solvent A (water) and solvent B (0.02% trifluoroacetic acid in acetonitrile) as follows: 0–20 min, 80% of A; 20–35 min, 40% of A; 35–50 min, 20% of A; 50–70 min, 10% of A, and 70–85 min, 0% of A at λ = 280 nm. The flow rate was 0.8 mL/min, and the injection volume was 10 µL. Polyphenolic compounds were detected by comparison of retention times with those of available pure standards.

### 4.4. Radical-Scavenging Activity Assays

#### 4.4.1. 2,2-Diphenyl-1-Picrylhydrazyl Radical-Scavenging Assay

The DPPH radical-scavenging assay used for the evaluation of free radical-scavenging activity of the LCBE was operated following the protocol described elsewhere [52]. Ascorbic acid was used as a positive control. The following equation was used to calculate the percent inhibition activity:

(1) $$\text{Radical-scavenging activity}\;(\%\;\text{inhibition})=\left[\frac{{\mathrm{Abs}}_{\mathrm{control}}-{\mathrm{Abs}}_{\mathrm{sample}}}{{\mathrm{Abs}}_{\mathrm{control}}}\right]\times 100$$

where Abs_control_ is the absorbance of the control and Abs_sample_ is the absorbance of the sample. All samples were analysed in three independent experiments.

#### 4.4.2. 2,2′-Azino-bis(3-ethylbenzthiazoline-6-sulphonic acid) Radical-Scavenging Assay

The method of Re et al. [53] was adopted for the ABTS radical-scavenging assay. The percent inhibition activity was calculated using Equation (1).

#### 4.4.3. Superoxide Radical-Scavenging Assay

The superoxide radical (O_2_^•^^−^) was generated by a non-enzymatic PMS/NADH system, which reduces nitro blue tetrazolium to purple-coloured formazan, and the O_2_^•^^−^-scavenging activity of the LCBE was measured according to a previously reported method [54]. Gallic acid was used as a positive control, and the percent inhibition activity was calculated using Equation 1.

#### 4.4.4. Hydroxyl Radical-Scavenging Assay

A previously described method [55] was adopted, with a minor modification, for the determination of the scavenging activity of the LCBE for hydroxyl radicals (HO^•^) generated by the Fenton reaction using a Fe^3+^-ascorbate–EDTA–H_2_O_2_ system. Equation 1 was used to determine the percent inhibition activity of the LCBE.

#### 4.4.5. Ferric Reducing Antioxidant Power Assay

The ferric reducing antioxidant power (FRAP) assay was performed to measure the reducing power capacity of LCBE as described previously [56] in which ascorbic acid was considered to generate the standard curve and calculate the antioxidant potentiality as an ascorbic acid-equivalent FRAP value (µM). 

#### 4.4.6. Cupric Reducing Antioxidant Capacity

The cupric reducing antioxidant capacity (CUPRAC) of the LCBE was determined according to the method described previously [57]. The ascorbic acid equivalent CUPRAC value (µM) was calculated from a standard curve generated for ascorbic acid.

#### 4.4.7. Oxygen Radical Absorbance Capacity 

The oxygen radical absorbance capacity (ORAC) assay was performed as previously reported [40]. Trolox, a water-soluble analogue of vitamin E, was used as a positive control and calculated the antioxidant potentiality as a trolox equivalent ORAC value (µM).

### 4.5. In Silico Toxic Risk Prediction and Biological Activity Spectrum

To estimate possible toxicity risks (mutagenicity, carcinogenicity, cardiotoxicity, skin irritant, hepatotoxicity and reproductive side effects) and the possible biological activities of the major compounds of LCBE, a computational simulation study was performed. For toxic risk prediction, four online computer programs: ACD/Labs (Toronto, ON, Canada), admetSAR server, pkCSM platform, and the PreADMET web-based application (http://preadmet.bmdrc.kr/) were employed. The toxic risks assessed were interpreted and expressed in a flexible manner: (+) low potential, (++) medium risk, (+++) high risk and non-detected risk (N) [58]. For biological activities spectrum prediction, PASS (available in http://www.pharmaexpert.ru/PASSonline/predict.php) was used. Prediction results were expressed in percentage of probable activity (Pa) and probable inactivity (Pi). Pa and Pi values vary from 0.000 to 1.000, thus, here, we considered only activities with Pa > Pi and Pa > 0.700.

### 4.6. Cell Culture and Cell Viability Assay

RAW 264.7 cells and Skin Fibroblast cells (BJ cells) (American Type Culture Collection, Manassas, VA, USA) were cultured at 37 °C in DMEM supplemented with 10% FBS, streptomycin–penicillin (100 µg/mL and 100 U/mL, respectively; HyClone, (St. Paul, MN, USA) in a humidified atmosphere of 5% CO_2_. The tetrazolium dye colorimetric test (MTT) was used to determine the viability of RAW 264.7 cells. RAW 264.7 cells (5 × 10^5^ cells/mL) and BJ cells (1 × 10^5^ cells/mL) were first cultured in 96-well plates for 24 h and treated with different concentrations of test samples, followed by removal of the media and addition of the MTT reagent to each well and incubation at 37 °C for 1 h. Then the media were also removed, and the wells were washed twice with PBS (pH 7.4). Then, 100% DMSO was used to dissolve the formazan and the absorbance in each well was measured at 570 nm using a microplate reader (Victor3, PerkinElmer, Waltham, MA, USA), and the percentage of viability was calculated.

### 4.7. Measurement of Intracellular ROS

Cellular oxidative stress due to ROS production by 2,2′-azobis(2-amidinopropane) dihydrochloride (AAPH) was measured spectrofluorometrically using the DCFH-DA method [59]. RAW 264.7 cells and BJ cells were first cultured in 96-well plates (5 × 10^5^ cells/mL) with DMEM for 24 h. Then, the cells were pretreated with various concentrations of the LCB extract. After 1 h, the cells were stimulated with AAPH (600 µM) and incubated for an additional 30 min. Consequently, they were washed with PBS twice and 25 µM DCFH-DA was added into the cells incubated for 30 min at 37 °C. The fluorescence intensity was calculated at an excitation wavelength of 485 nm and an emission wavelength of 528 nm using a Victor3 fluorescence microplate reader (PerkinElmer). 

### 4.8. Reverse Transcription-Polymerase Chain Reaction 

TRIzol (Invitrogen, Carlsbad, CA, USA) was used to extract the total RNA using manufacturer’s protocol. Total RNA (2 µg) was reverse transcribed using reverse transcriptase (MP Biomedicals, Santa Ana, CA, USA) and oligo(dT) primers. cDNA was synthesized using a thermal cycler Dice TP600 (Takara Bio, Inc., Otsu, Shiga, Japan). Polymerase chain reaction (PCR) products were visualised by ethidium bromide staining after electrophoresis. Specific oligonucleotide primers for mouse transcripts were shown in Table 3.

### 4.9. Preparation of Cell Lysates and Western Blotting

Standard protocol was used to prepare the RAW 264.7 cell lysates and then mixed with sodium dodecyl sulphate (SDS) sample buffer (250 mM Tris–HCl, pH 6.8, 0.5 M dithiothreitol, 10% sodium SDS, 0.5% bromophenol blue, 50% glycerol, 5% 2-mercaptoethanol), followed by denaturing at 100 °C for 5 min. For nuclear protein extraction, a nuclear/cytosolic fractionation kit (Cell Biolabs, Inc., San Diego, CA, USA) was used. SDS–polyacrylamide gel electrophoresis (10%) was performed to separate the sample protein (20 µg/lane). Following electrotransfer to nitrocellulose membranes (Whatman, Dassel, Germany), the membranes were incubated overnight with 5% skim milk used to block the protein, followed by incubation of the membranes incubated overnight with primary antibodies such as anti-goat IgG–horse radish peroxidase (HRP; Santa Cruz Biotechnology), and anti-rabbit IgG–HRP (Santa Cruz Biotechnology) applied as secondary antibodies. The antigen–antibody reaction was exposed using an enhanced chemiluminescence system (PerkinElmer).

### 4.10. Statistical Data Analyses

All data are denoted as the mean ± standard deviation. Data were analysed using one-way analysis of variance. Differences were considered significant at *p* < 0.05 or *p* < 0.01. All analyses were executed using SPSS for Windows, version 10.07 (SPSS, Chicago, IL, USA).

## Figures and Tables

**Table 1:** Toxicity prediction of major identified constituents of *Lannea coromandelica* Bark extract (LCBE) obtained by computer simulation.

ID Substances	Toxic Risk by ACD/Labs ^1^; admetSAR ^2^; pkCSM ^3^; PreADMET ^4^					
Phytoconstituents	Mutagenic	Carcinogenic	Cardiotoxic	Skin Irritant	Reproductive System Toxicity ^1^	Hepatotoxic
Gallic acid	N^1^,		N^1^,	N^3^,	N^1^	N^3^
	N^2^,	N^2^,	(+)^2^,			
	N^3^,	N^4^,	N^3^,			
	(+)^4^		(+)^4^			
EGCG	(+)^1^,		(+)^1^,	N^3^	N^1^	N^3^
	N^2^,	N^2^,	(+)^2^,			
	N^3^,	(+)^4^,	N^3^,			
	(+)^4^		(++)^4^			
Cathechin	(+)^1^,		(+)^1^,	N^3^	(+)^1^	N^3^
	N^2^,	N^2^,	(+)^2^,			
	N^3^,	N^4^,	N^3^,			
	(+)^4^		(++)^4^			
Chlorogenic acid	(+)^1^,		N^1^,	N^3^	N^1^	(+)^3^
	N^2^,	N^2^,	(+)^2^,			
	N^3^,	(+)^4^,	N^3^,			
	N^4^		(+++)^4^			
Caffeic acid	(+)^1^,		N^1^,	N^3^	N^1^	N^3^
	N^2^,	N^2^,	(+)^2^,			
	N^3^,	(+)^4^,	N^3^,			
	(+)^4^		(++)^4^			

The scale of toxicity risk ranges from low (+), medium (++), high (+++) and none (N). EGCG: epigallocatechin-3-gallate. Superscript numerical number represent the online computer programming system which is being used to predict the toxicity (see for details in materials and methods). N^1^, N^2^, N^3^, and N^4^ represent no toxicity was found from the respective online computer programming system.

**Table 2:** Pharmacological activities predicted for the major identified chemical constituents of *Lannea coromandelica* Bark extract (LCBE).

Phytoconstituents	Main Predicted Properties by PASS Online	Pa ^#^	Pi ^#^
Gallic acid	Antiseptic	0.921	0.003
	Astringent	0.884	0.001
	Antiinfective	0.828	0.005
	Fibrinolytic	0.823	0.004
	Mucomembranous protector	0.814	0.015
	Mucositis treatment	0.798	0.014
	Kidney function stimulant	0.779	0.004
	HO-1 expression enhancer	0.732	0.005
EGCG	HMOX1 expression enhancer	0.985	0.001
	Mucomembranous protector	0.950	0.003
	Fibrinolytic	0.942	0.003
	Free radical scavenger	0.934	0.001
	Astringent	0.927	0.001
	Antioxidant	0.827	0.003
	Cardioprotectant	0.822	0.003
	Hepatoprotectant	0.820	0.004
	Antihemorrhagic	0.794	0.002
	Antiviral (Influenza)	0.771	0.003
	Antihypercholesterolemic	0.705	0.008
Catechin	HO-1 expression enhancer	0.971	0.001
	Mucomembranous protector	0.962	0.003
	Fibrinolytic	0.959	0.002
	Antihemorrhagic	0.926	0.002
	Astringent	0.921	0.001
	Free radical scavenger	0.850	0.002
	Antioxidant	0.828	0.003
	Chemopreventive	0.791	0.004
	Hepatoprotectant	0.769	0.005
Chlorogenic acid	Choleretic	0.920	0.001
	Free radical scavenger	0.856	0.002
	Antioxidant	0.809	0.003
	Antieczematic	0.808	0.017
	Antineoplastic	0.785	0.014
	Mucomembranous protector	0.752	0.034
Caffeic acid	Mucomembranous protector	0.945	0.003
	Mucositis treatment	0.873	0.008
	HO-1 expression enhancer	0.799	0.004
	Antiseptic	0.782	0.004
	Vasoprotector	0782	0.006
	Fibrinolytic	0.750	0.009
	Cytoprotectant	0.722	0.039
	Antieczematic	0.702	0.005

^#^ Pa: probable activity; Pi: probable inactivity; HO-1: heme oxygenase 1.

**Table 3:** List of primers used in this study.

Gene Name	Sequences	
*Sod1*	Forward	AAGCGGTGAACCAGTTGTGT
	Reverse	GCCAATGATGGAATGCTCTC
*Sod2*	Forward	AACTCAGGTCGCTCTTCAGC
	Reverse	CTGTAAGCGACCTTGCTCCT
*Gpx1*	Forward	ACACCGAGATGAACGATCTG
	Reverse	ATGTACTTGGGGTCGGTCAT
*Cat*	Forward	CACCCACGATATCACCAGATAC
	Reverse	GAAGACTCCAGAAGTCCCAGAC
*Hmox1*	Forward	ACGCATATACCCGCTACCTG
	Reverse	TCCTCTGTCAGCATCACCTG
*Gclc*	Forward	GGAGGCGATGTTCTTGAGAC
	Reverse	GGGTGCTTGTTTATGGCTTC
*Gclm*	Forward	AGTTGCACAGCTGGACTCTG
	Reverse	TCGGGTCATTGTGAGTCAGT
*Nqo1*	Forward	CTGGCCCATTCAGAGAAGAC
	Reverse	GTCTGCAGCTTCCAGCTTCT
*Nrf2*	Forward	ACATCCTTTGGAGGCAAGAC
	Reverse	GGGAATGTCTCTGCCAAAAG
*Gstpi*	Forward	GCCCAGATGGATATGGTGAA
	Reverse	ATGGGACGGTTCACATGTTC
*Keap1*	Forward	GCTACAACCCCATGACCAAC
	Reverse	GCGGAGTTAAGCCGGTTAGT
